# Analysis of the Deburring Efficiency of EN-AW 7075 Aluminum Alloy Parts with Complex Geometric Shapes Considering the Tool Path Strategy During Multi-Axis Brushing

**DOI:** 10.3390/ma17246267

**Published:** 2024-12-21

**Authors:** Jakub Matuszak, Andrzej Kawalec, Michał Gdula

**Affiliations:** 1 Department of Production Engineering, Mechanical Engineering Faculty, Lublin University of Technology, Nadbystrzycka 38D, 20-618 Lublin, Poland; 2 Department of Manufacturing Techniques and Automation, Faculty of Mechanical Engineering and Aeronautics, Rzeszów University of Technology, Wincentego Pola 2, 35-959 Rzeszów, Poland; ak@prz.edu.pl (A.K.); gdulam@prz.edu.pl (M.G.)

**Keywords:** deburring, brushing, lead angle, five-axis milling, tool path strategy

## Abstract

The paper presents the results of an analysis of the effect of brushing on the edge condition of workpieces with complex geometric shapes, formed during milling, on a five-axis DMU 100 monoBLOCK machining center. A set of EN-AW 7075 aluminum alloy specimens with curvilinear edges requiring multi-axis machining was prepared. The change of edge condition after the milling process was realized using Xebec tools with flexible ceramic fibers. The effects of brush fiber type and parameters related to tool design were analyzed. Different brushing strategies were employed on the five-axis machining center. It was shown that, for curvilinear edges, there were different effects for concave and convex edges depending on the employed tool strategy, including the type of tool, its configuration, and its orientation towards the workpiece. For a lead angle of β = 0°, the machined edge was characterized by variable chamfer widths, in spite of maintaining other machining parameters constant. The use of a lead angle β > 0 produced a stable edge with repeatable characteristics. The range of fiber interaction increased with increasing the lead angle and fiber working length.

## 1. Introduction

Aluminum alloys are lightweight materials that are widely used in the aerospace and automotive industries because of their high strength-to-weight ratios. The effort to minimize the weight of the finished product results in a large portion of the material volume being removed in the manufacturing process. Up to 90% of the material is converted into chips, usually by milling. The manufacture of pocket structures with complex geometric shapes requires the use of multi-axis machines to make the complex-shaped component within a single fixture. The complex geometric shape makes the final product have a large number (and total length) of edges. Owing to the continuous development of cutting tools (geometry, tool materials, coatings), it is possible to do the finishing of workpieces, even in the hardened state, through milling. Therefore, milling may be the last operation in the manufacturing of the final product.

During milling, burrs may appear on each of the newly formed edges. The burr formation phenomenon after machining is very common and is related to the formation of chips [1,2]. When the tool blade exits the cutting zone reaching the edge of the workpiece, burrs may be formed due to the plastic flow of the material. Strong elastic and plastic deformations occur when the cutting layer is separated and transformed into a chip. Plastic deformations within the edge of the workpiece initiate the process of burr formation. A burr is a deformed piece of the material deviating from the theoretical shape of the edge, which has not been separated from the workpiece in the form of chips. The process of burr formation during orthogonal cutting has been investigated in many scientific studies [2,3,4]. Burrs can be classified depending on their shape or the type of machining applied. Gillespie et al. [5] presented one of the first classifications related to the mechanism of burr formation, dividing burrs into four groups. In turn, Lin proposed the classification of burrs based on their shape [6]. Much attention has been paid to the formation of burrs during drilling [7,8,9,10,11]. This is because, due to the nature of drilling, it is impossible to eliminate burrs when the drill makes a through hole. Classification of burrs formed during drilling refers to the shape, formation mechanism, location, and size of the burrs. Factors affecting the above-mentioned burr parameters include the chisel edge, the value of the included angle, the chip groove helical line, the secondary cutting edge angle, material properties, machining parameters, and tool wear [8,11,12].

Burrs can be problematic for parts in a moving assembly. During cutting, most of the generated heat is dissipated with the chips. The impact of the tool on the workpiece material can cause changes in the material properties. The cutting temperature can lead to chip hardening. If such chips are not separated from the workpiece material, they may form burrs that are harder (e.g., due to hardening) than the workpiece material. Such hard and brittle material poses a very high risk in structures where holes are used to transport the medium. There have been reports of burrs appearing in fuel injector holes, which could lead to engine damage [13].

Previous studies of burr formation led to the development of methods for burr minimization. It is better to minimize burrs at the machining stage [14,15,16], rather than to remove them in subsequent operations. Cutting tool manufacturers often provide recommendations related to machining strategies. The operation of a cutting tool can be divided into three phases: input, the passage of the tool through the material, and output. Since the exit of the material is the most sensitive phase, it is important to keep the cutter constantly engaged. In addition, climb milling is recommended, as it leads to a reduction in burr height. When the machining process is performed on a multi-axis center, the situation becomes more complex. In addition to the typical technological parameters and machining strategies on multi-axis machines, the lead and tilt angles must also be taken into account [17]. Gdula and Mrówka-Nowotnik analyzed different machining strategies in relation to tool wear and chip formation [18]. The study was conducted using varying tool axis orientation parameters: the lead angle range was 1.37–20° and the tilt angle range was −18.86–20°. Innovative machining strategies can also prove useful when generating NC paths in CAM software. Zaleski et al. [19] analyzed the effect of the machining strategy used on the passive force, cutting torque, machining efficiency, surface topography, and chip shape in aluminum alloy milling. The study involved comparing the classic strategy available in NX with the iMachining technique.

The total cutting force F and the roughness parameters Ra and Rz of machined surfaces as well as the relationships between specified cutting parameters and the analyzed roughness parameters were investigated by Szajna et al. [20]. A clear relationship between the total cutting force and the roughness parameters was established. The correct separation of a cutting layer is also related to the tool precision. Żurawski et al. [21] analyzed the run-out phenomenon in the milling process of 40 HM steel, Al7035 aluminum alloy, and Ti Grade 5 titanium alloy. It was found that a statistical approach should be employed to design correct regression models of the observed relationships. Gancarczyk et al. [22] showed that the application of information criteria and the adjusted coefficient of determination were helpful in improving the regression models of experimentally observed relationships between selected parameters.

Burrs formed on edges should be removed to prevent any technological, construction, or operational safety problems. The edge quality of machined parts is important for many reasons. The removal of burrs makes it easier or even possible to perform assembly and control operations or subsequent technological operations on the workpiece. In addition, burrs and sharp edges are dangerous to both assembly line operators and users of finished products. In order to ensure the proper operation of moving parts in an assembly, edges should be characterized by a specific, defined state. The desired edge states are chamfered, rounded, or free of burrs. There are also burr characteristics that determine the ease of their removal. The first one is the type of connection to the workpiece material, which is determined by the properties of the material. Another aspect is the location of burr formation. Burrs are most easily removed from the outer and easily accessible edges. It is more difficult to remove burrs from edges that are formed where surfaces create an obtuse angle with difficult access. The most difficult-to-remove burrs are located on edges in deep blind holes, on concave surfaces, and inside corners. The third major factor affecting the ease of deburring is burr height.

There are many methods of deburring. Among the most common are hand deburring, grinding, and milling with file tools. It is also possible to remove burrs from objects placed in an abrasive medium. These include rotary abrasion, abrasive flow machining (AFM), and abrasive blasting [13]. Depending on the degree of automation, the deburring process can be divided into manual, semi-automatic, and automatic processing.

Manual deburring is mainly used in single-piece production. However, in certain situations, such as large-sized and complex-shaped items (aerospace industry), it is also used in mass production. During manual deburring, it is difficult to eliminate cases of deeper unintentional edge cuts, which could not only reduce the aesthetic value of the finished product but also create a notch with stress concentration.

A more efficient process is semi-automatic machining using belt grinders or hand-held high-speed tools. Edge machining on belt grinders is used for small-sized workpieces in single-piece production. In contrast, machining with high-speed tools is used for larger-sized workpieces, where it is often easier to make precise movements with a hand grinder along the edges of workpieces.

Taking into account economic aspects, automated machining is the most desirable process. A widely used group of automated methods are those in which the job of smoothing edges and removing burrs is performed by an abrasive medium. This includes vibratory machining, abrasive flow machining, and chemical, electrochemical, or electrical discharge machining [23,24,25,26,27,28,29,30,31,32]. Burrs can also be removed by the thermal method of burning with heat energy.

There also exist many ways to remove burrs via typical machining operations, such as milling. Most ways of removing burrs through typical machining methods involve applying a rigid tool to the workpiece and performing a chamfer. However, there is a possibility that the newly created edge resulting from chamfering with rigid tools will be sharp or have “micro-burrs” on it, which can be undesirable when making precision parts.

One simple and inexpensive method, with the possibility of automation, is brushing. Thanks to rotating brushes with flexible fibers, a specific edge condition can be achieved after machining. Brushing tools are used in machining processes for, among other things, deburring, edge rounding, glossing, and surface cleaning, as well as for removing surface defects [33,34]. In addition, brushing can be used to produce a specific pattern of structure texture for a decorative effect; it can precede the joining process or be used to generate specified properties of the surface layer [35]. The impact of fibers on the machined surface produces similar effects in the surface layer as those achieved by shot peening or burnishing [36,37]. Brushing is also used to remove surface defects [38], which can be detected by recurrence analysis [39]. In addition, brushes are easy to use in automated machining because the flexible ends of the filaments performing the machining operation easily adapt to the machined surface, without a need for tool positioning and measuring systems [40,41,42,43]. The widespread use of brushes results from their good performance, the ease of implementation in manual or automatic surface finishing, and uniform distribution of cutting forces on the surface of the workpiece [33,44,45]. This allows for controlled material removal and simple workpiece mounting and reduces the risk of damage to both the machine and the workpiece.

The use of ceramic brushes on CNC machining centers allows the deburring of large workpieces to be carried out automatically by planning the tool path along the edges of these workpieces. Taking advantage of the fact that the workpiece is mounted on the machine table, the deburring process can be performed using brushes.

Previous studies investigating the effect of brushing on edge state have mainly focused on the impact of technological cutting parameters on the condition of a straight edge. In contrast, this study investigates the effect of the lead angle on the condition of a curvilinear edge, which is novel compared to previous studies related to the deburring process by brushing. The investigation required the use of a five-axis milling center to position the tool in relation to the complex shape of the workpiece edge in the way assumed in the research plan.

## 2. Materials and Methods

### 2.1. Material and Shape of the Samples

Test samples were made of AW 7075-T651 aluminum alloy owing to its high strength, machinability, and corrosion resistance. These properties make this alloy suitable for use in the production of components for the automotive and aviation industries. The chemical composition and physical properties of AW 7075-T651 are listed in Table 1.

**Figure 1:**
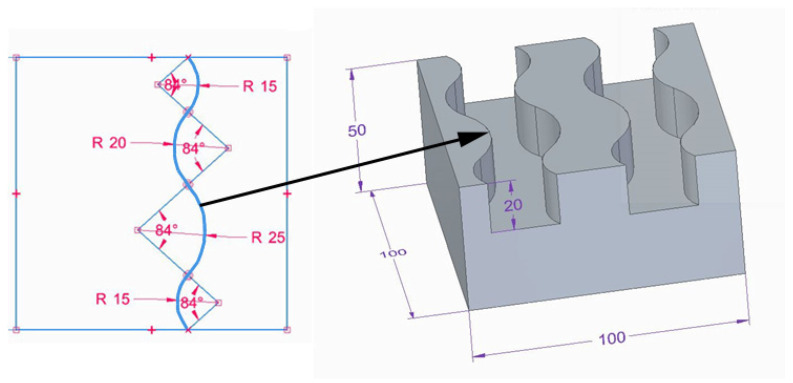
Model of a workpiece with curved edges.

A model of the sample was prepared, assuming that there were four edges on each sample. Each edge had four curves of a constant radius, two concave (R15 and R20) and two convex (R15 and R25), in order to determine the edge curvature effect on machining results. The model is shown in Figure 1.

To create complex edges, 20 mm deep curvilinear grooves were made in cuboid samples with the dimensions 100 × 100 × 50 mm.

### 2.2. General Methodology

**Figure 2:**
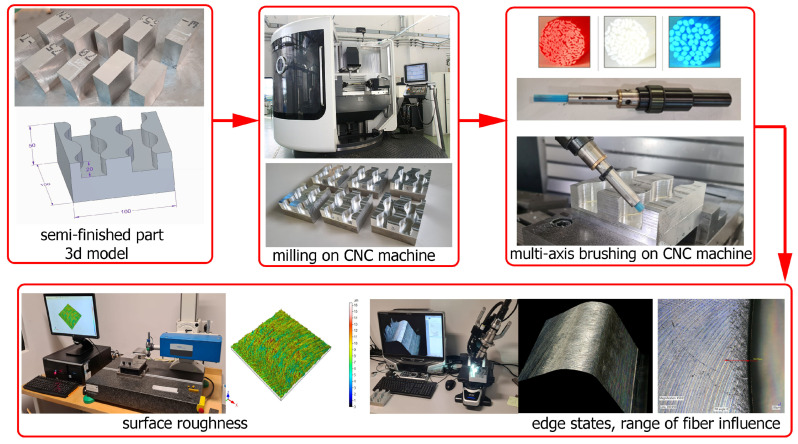
Experimental setup.

An experimental setup is shown schematically in Figure 2. The brushing process was carried out on a multi-axis milling center, the DMU 100 monoBLOCK.

**Figure 3:**
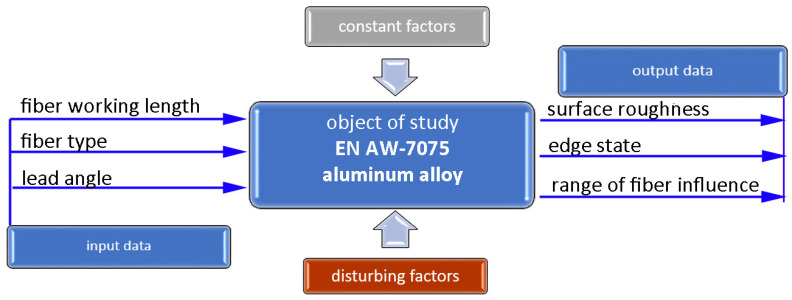
Schematic diagram of the research plan.

First, a model of the workpiece was created to study the effect of brushing on the edge condition using a multi-axis machining center. After that, the milling process was carried out with constant technological cutting parameters in order to maintain the repeatability of the shape and quality of the edges of the test samples. The next stage was to design experiments with the Statistica 13.3 software (DOE). The response surface methodology (RSM) based on a rotatable design with k = 2 was adopted, in which A was the length of fiber protruding from the sleeve and B was the lead angle. A schematic diagram of the study is shown in Figure 3. The output factors were the surface roughness on flat surfaces achieved in accordance with the adopted experimental plan, the edge states after brushing, and the range of fiber impact on the surfaces located in the area of the edge. The following factors were maintained constant: the technological parameters of brushing (rotational speed, feed speed, and depth of cut), tilt angle, and orientation of the workpiece with relation to the tool axis. Depending on the brush type, the manufacturer provides the maximum permissible and recommended rotational speeds. The recommended speed was assumed in the tests at *n* = 8000 rpm. In turn, the cutting depth a_p_ = 0.5 mm and feed rate v_f_ = 500 mm/min were selected, taking into account the recommendations related to the types of burrs and the expected surface quality. G-codes were generated in NX 12.0 using the CAM module for both milling and brushing. The experiment was carried out on a five-axis DMU 100 monoBLOCK center.

Before starting the main experiment, a brushing operation was carried out with the tool axis set perpendicular to the tangent to the curve at the contact point. At the same time, the tool moving along the edge had its lead and tilt angles set to zero. The process of brushing edges and flat surfaces was independently carried out to assess the effect of brushing conditions on the roughness and condition of the edges. The result of the research was the assessment of the surface roughness and edge condition as chamfer or rounded after brushing.

### 2.3. Tools

Prior to brushing, all specimens were subjected to milling to produce parts with complex geometric shapes. The milling operations were carried out with an MA Ford Europe—137VR 20N3-1.0R carbide milling cutter (diameter D = 20 mm). The milling parameters were maintained constant (v_c_ = 500 m/min, f_z_ = 0.1 mm/tooth, a_p_ = 5 mm) in order to ensure that the edges of the surfaces exhibited constant states (repeatable burr height). After that, the produced edges were examined to determine the effect of brushing on the edge states and the surface roughness in relation to milling.

**Figure 4:**
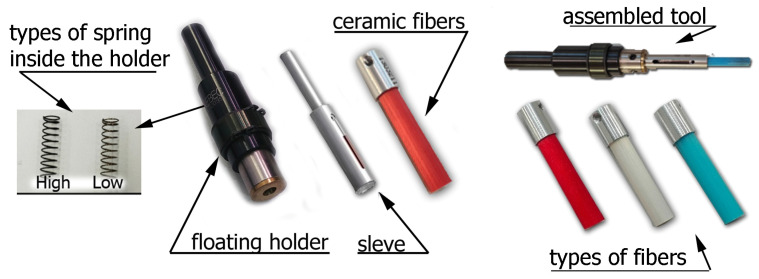
Ceramic brush design.

The Xebec brushes used in the study are shown in Figure 4. The sleeve was mounted in a special floating holder. Two types of springs could be mounted in the holder, each exerting a different pressure on the surface being processed. Ceramic fibers were mounted inside the sleeve, allowing them to be extended from 5 to 30 mm, which significantly affects the efficiency of the machined surfaces and edges.

Three types of ceramic fibers were used in the tests: red, which are flexible and used for soft materials; white, which are rigid and provide higher efficiency in deburring and reducing surface roughness; and blue, which have the highest stiffness among the fibers used in the tests and are used to machine difficult-to-cut materials. The condition of the brush fibers was monitored during the experiment. In order to avoid the influence of tool wear on the results, the fibers were replaced with new ones when wear was detected.

### 2.4. Brushing Conditions

**Figure 5:**
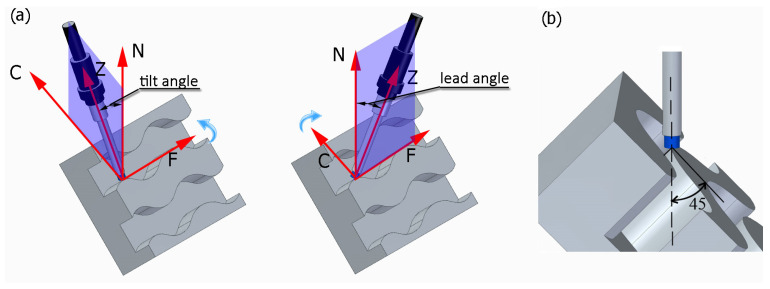
Tool setting in the experiment: (**a**) visualization of tilt and lead angles, (**b**) workpiece orientation.

Fixed technological parameters of brushing were used in the study. The rotational speed was set at *n* = 8000 rpm, the feed rate along the machined curved edge was v_f_ = 500 mm/min, and the depth of cut was a_p_ = 0.5 mm. Figure 5a shows the tilt and lead angles. The post-processor converts the tilt and lead angles, and the CNC machine controller interprets these angles as inclination and rotation. The value of the tilt angle was fixed at 0 degrees. The sample was positioned with reference to the tool axis in such a way that both faces forming an edge were at an angle of 45 degrees to the tool axis (Figure 5b). The study used variable lead angles β and variable fiber working length W (Table 2).

**Figure 6:**
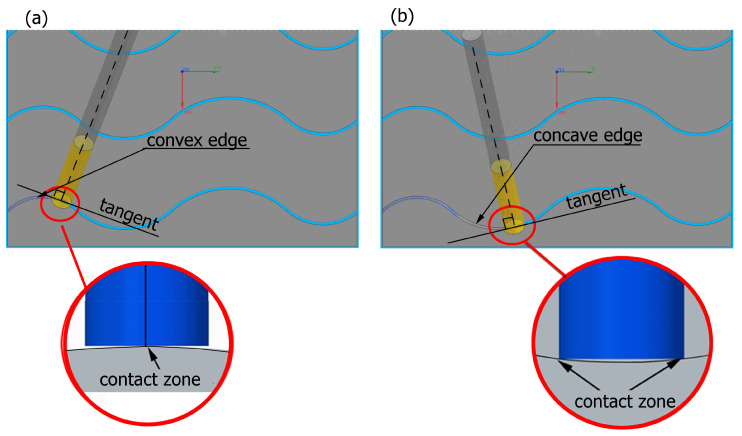
Tool contact during brushing with a lead angle of β = 0° on (**a**) convex edge, (**b**) concave edge.

Preliminary tests were conducted with the assumption that the lead angle was β = 0. In addition, the tool axis was set perpendicular to the tangent to the curve at the point of contact, as illustrated in Figure 6.

With this setting, the conditions of contact between the tool and the brushed edge would change depending on whether a concave or convex edge was machined. When machining the convex edge, the flat brush face was in contact with the workpiece near the tool axis. On the concave edge, in contrast, the contact occurred in the area of the outer diameter of the brush. Thus, the cutting speed at the point of contact between the tool and the workpiece changed, and thus the machining effects along the machined edge were different.

**Figure 7:**
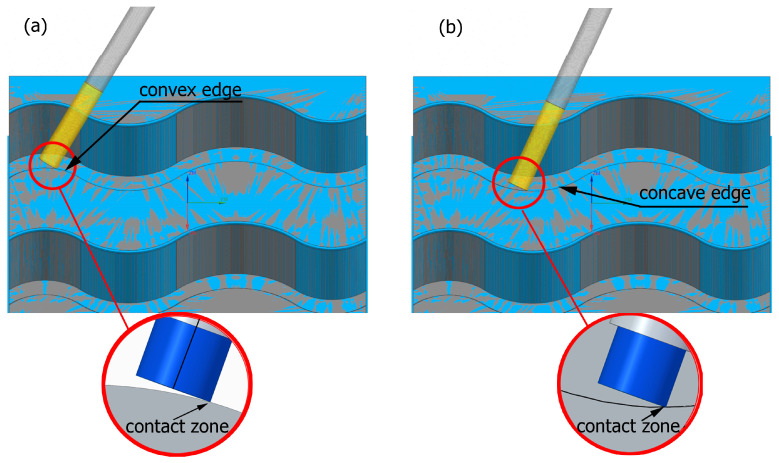
Tool contact during brushing with a lead angle of β > 0° on: (**a**) convex edge, (**b**) concave edge.

The main experiment was conducted using variable lead angles. This allowed the tool contact to be maintained in the area of the brush outer diameter regardless of the curvature of the edge (Figure 7).

**Figure 8:**
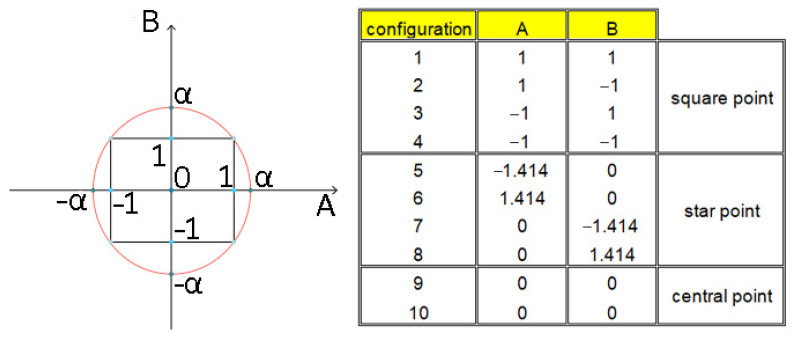
Schematic view of rotatable central composite designs.

The objective of the main experiment was to determine the response surface using a rotatable design (Figure 8).

The value assigned to the star point was determined based on

(1) α=(nc)14

where:

*n_c_*—the number of square points

The brush construction allowed us to set the fiber working length in the range of 0–30 mm. The central point was set at 15 mm. The iteration was set at 7 mm; therefore, the square point (B = −1 and B = 1) values were 22 and 8 mm, respectively (central point value ± iteration). The length of the diagonal of a square d = $a\sqrt{2}$ with a side of a = 14 mm (a = 22 mm–8 mm) is d = 19.8 mm; therefore, the star point values were set at 5.1 and 24.9 mm (central point value ± d/2). The values of square, star, and central point for lead angles were determined in the same way, while the lead angle range was determined on the basis of preliminary tests. Table 2 lists the values of the lead angle β and the fiber working length W (according to rotatable design experiment plan) used in the experiment.

### 2.5. Measurements Methodology

**Figure 9:**
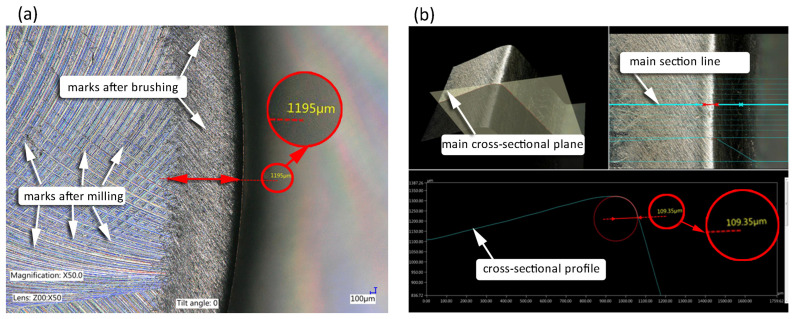
Methodology of determining (**a**) the range of fiber influence, (**b**) edge state.

Surface roughness was evaluated using a T8000RC 120–140 device from Hommel Etamic (Jena, Villingen-Schwenningen, Germany). The 3D surface topography visualization was performed on square surfaces with the dimensions 4.8 × 4.8 mm^2^. Edge condition was examined with a Keyence VHX 5000 digital microscope (Keyence Ltd. HQ & Laboratories, Osaka, Japan). Figure 9 shows the methodology for determining the edge state and the range of fiber impact on the surfaces located in the edge area.

The depth composition, combined with the functionality of the software, allows the design of a 3D model in which measurements can be made on cross-sectional profile.

## 3. Results

### 3.1. Surface Roughness

Since fibers have a range of influence on surfaces that are close to the machined edge, the surface roughness changes in this area. Maintaining the roughness specified in the technological documentation is very important in parts produced in various industries. The most desirable effect would be to remove burrs and improve, or maintain, the surface quality at the level of machining before brushing. Table 3 shows the surface topography after milling and after brushing with arithmetic mean height Sa. The characteristic periodic pattern of milling marks resulting from the kinematic–geometric representation of the blade outline in the workpiece can be seen.

**Figure 10:**
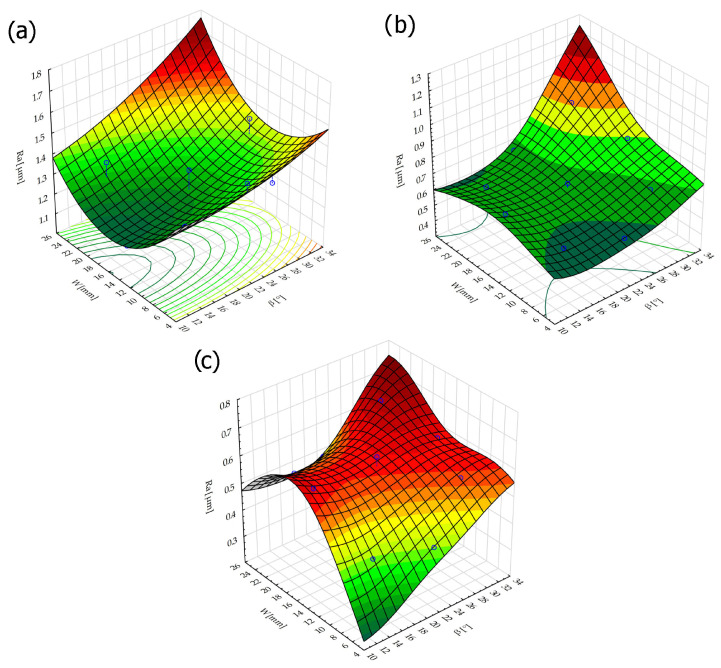
Effects of lead angle and fiber working length on surface roughness for the different fibers: (**a**) blue (high stiffness), (**b**) white (medium stiffness), (**c**) red (low stiffness).

The brushing process removed the tops of the micro irregularities. Brushing with the blue fibers (high stiffness) and white (medium stiffness) caused an increase in roughness compared to that obtained in milling, which may indicate that the fibers impacted too much on the machined surface. For the red fibers (low stiffness), the roughness remained at a level similar to that in milling. The surface response for a rotatable central composite design is shown in Figure 10. For the white (medium stiffness) and red fibers (low stiffness), the lowest roughness values were obtained for the smallest fiber working length W and lead angles β. As the values of the lead angle β and the fiber working length W increased, the value of the roughness parameter Ra increased too. This is because the centrifugal force caused bending perpendicular to the axis of the brush fibers. However, the depth of cut a_p_ = 0.5 mm was determined and applied in the NC programs for undeformed fibers. As the fiber advanced and the lead angle increased, the actual depth of fiber cutting (the distance between the work surface and the furthest point of freely rotating fibers measured perpendicular to the surface) increased too, which made the fiber interaction with the machined surface more intense.

For the red fibers (low stiffness) at small lead angles in the 15 to 29 mm extension range, an inflection point appeared and the roughness became lower (it was comparable to that after milling) because the highly flexible fibers would bend after impacting the work surface, which reduced the intensity of the impact.

The lowest surface roughness was obtained for the red fibers (low stiffness) using the smallest lead angle and the smallest fiber working length.

The regression model for the blue fibers (high stiffness) with an *R*^2^ = 0.68 coefficient of determination informing the quality of the model fit is as follows:

(2) $$Ra=1.7338-0.011*\beta +0.0003*\beta^{2}-0.0661*W+0.002*W^{2}+0.0004*\beta *W$$

where:

*β*—lead angle
*W*—fiber working length

The regression model for the white fibers (medium stiffness) with the highest coefficient of determination, amounting to *R*^2^ = 0.88, is as follows:

(3) $$Ra=1.01-0.043*\beta +0.0009*\beta^{2}-0.0067*W-0.0002*W^{2}+0.001*\beta *W$$

In turn, for the red fibers (low stiffness) with a value of *R*^2^ = 0.73, the regression model is as follow:

(4) $$Ra=0.217-0.0044*\beta +0.0002*\beta^{2}+0.043*W-0.0012*W^{2}-0.00009*\beta *W$$

### 3.2. Edge States

**Figure 11:**
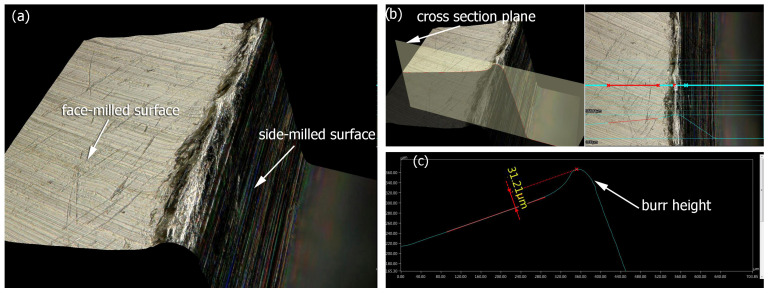
Top burrs after milling: (**a**) model composition, (**b**) cross section selection, (**c**) burr height measurement.

After the milling process, top burrs formed on the edges of the milled samples, which demonstrates the necessity of further treatment to change the unfavorable edge condition. The height of the top burrs was measured using a digital microscope due to the model composition (Figure 11a) and then a place on the model was selected where a cross-section had been created (Figure 11b), from where the burr height was measured (Figure 11c). This type of burr is created as a result of the separation of the cut layer by the side of the mill. The average burr height for all milled samples was 30 µm.

Table 4 shows the edge states after the brushing process for both concave and convex edges, where the lead angle was β = 0. The use of this lead angle causes the brush face interacting with the edge to form a characteristic chamfer (a flat surface in place of the previously visible burr). The transition at the point of inflection between the concave and the convex edge results in a change in the nature of the contact between the tool and the machined edge. A significant difference in the width of the chamfer depending on the curvature of the edge can be observed, which is not beneficial in terms of the stability and uniformity of the edge.

**Figure 12:**
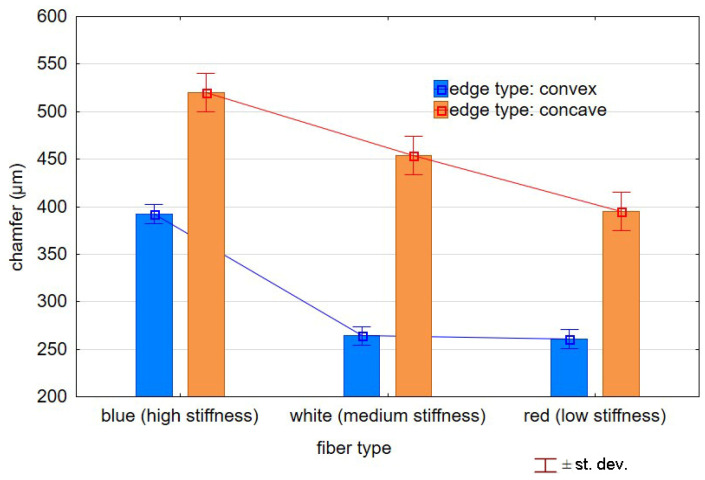
Effect of edge type on chamfer width.

Figure 12 shows the effect of edge curvature on the chamfer value for different fibers. The significant differences in the chamfer width along the edge indicate the need to change the brushing strategy to stabilize the edge state. The differences in chamfer width depending on the edge types (convex or concave) ranged from 30 to over 70%. The whiskers shown in the graph reflect the standard deviations of the results for particular edge types and brush stiffnesses used in the brushing process.

The use of lead angles β > 0 made the edge state uniform regardless of the curvature of the edge. Table 5 summarizes the edge states obtained in the experiment. Since two types of edge states were obtained for the blue fibers, i.e., chamfered and rounded, response surfaces are shown for the white and red fibers.

**Figure 13:**
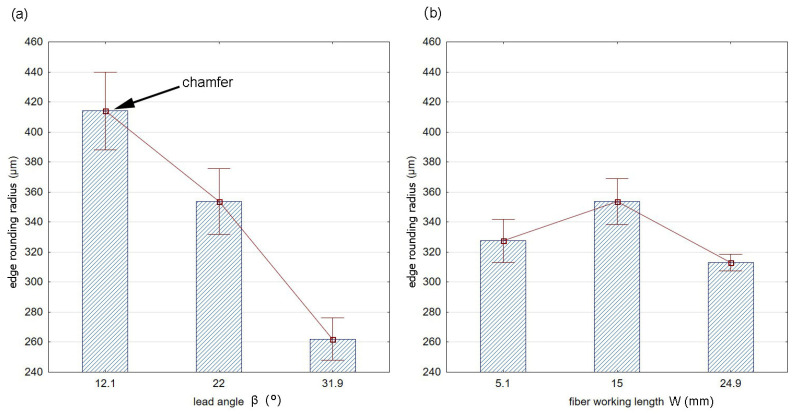
Effect of (**a**) lead angle β (for constant W = 15 mm), (**b**) fiber working length W (for constant β = 22°) on the value of the rounding radius for the blue fibers.

Figure 13 shows the effects of the fiber working length W (Figure 13a) and the lead angle β (Figure 13b) on the value of the rounding radius for the blue fibers. The whiskers marked in the diagram indicate the standard deviations of the results for particular lead angles and fiber working lengths used in the brushing process.

There was a visible decrease in the radius as angle β increased. This is due to the fact that the larger the lead angle is, the less the working part (ends of the fibers) is used, because even though the entrance to the contact zone occurs on the surfaces forming the edge, it is at a greater distance from the machined edge.

**Figure 14:**
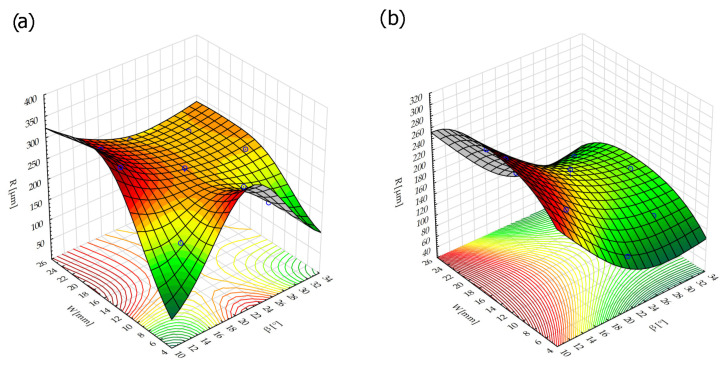
Response surface: (**a**) white fibers (medium stiffness), (**b**) red fibers (low stiffness).

The response surface describing the effect of the tested factors on the edge rounding radius for the white (medium stiffness) and red (low stiffness) fibers is shown in Figure 14.

The rounding radius regression model for the white fibers (medium stiffness) with a coefficient of determination of *R*^2^ = 0.25 is as follows:

(5) $$R=328.86-7.38*\beta +0.098*\beta^{2}+1.47*W-0.011*W^{2}$$

The low *R*^2^ value indicates a low quality of fit of the tested model to the data.

The regression model for red fibers (low stiffness) with the highest value of coefficient of determination, *R*^2^ = 0.93, is as follows:

(6) $$R=314.3-19.69*\beta +0.309*\beta^{2}+19.05*W-0.612*W^{2}+0.013*\beta *W$$

### 3.3. Range of Fiber Influence

**Figure 15:**
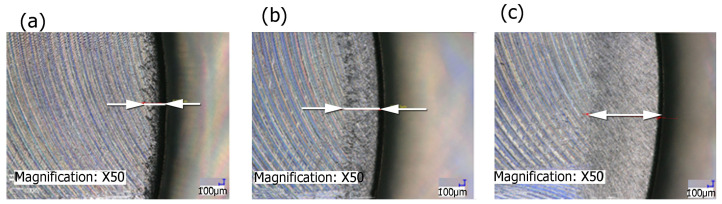
Range of fiber impact on surfaces forming an edge for different fibers: (**a**) blue, (**b**) white, (**c**) red.

Figure 15 illustrates the range of fiber impact on surfaces forming an edge. The range of fiber influence is the distance measured on the surfaces forming an edge, determined from the edge of the sample (from which the burrs have been removed) to the end of the visible marks and scratches left by the fibers during the edge deburring process. A characteristic feature of the fibers used in the study was their stiffness, related to the different cross-sectional area of a single fiber. Red fibers had the smallest cross-sectional area, while blue fibers had the largest. This resulted in differences in the deformation of the fibers under the influence of both cutting forces and centrifugal force from the rotary motion of the tool. For the lower stiffness of fibers, their deformation under the action of centrifugal force was greater, which for large lead angles increased the range of impact. The intense degree of impact of the fibers hitting surfaces that formed an edge resulted in the removal of marks caused by the milling cutter blade on the surfaces.

**Figure 16:**
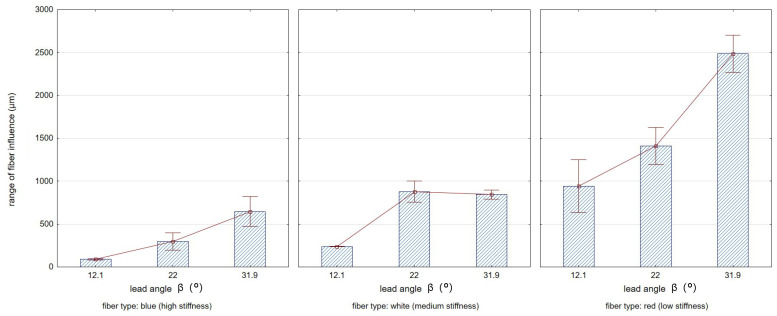
Effect of the lead angle β on the range of fiber influence on the surfaces forming the edge (for constant W = 15 mm).

**Figure 17:**
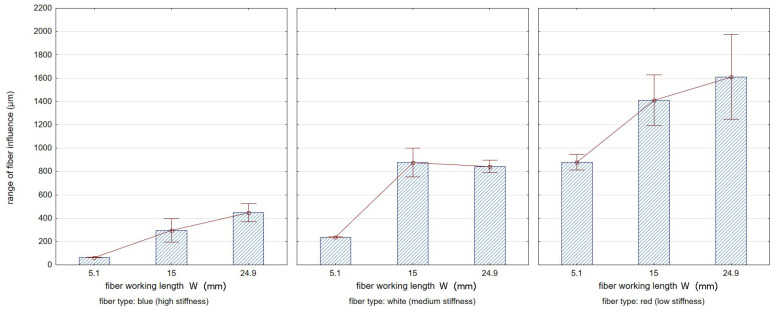
Effect of the fiber working length on the range of fiber influence on the surfaces forming the edge (for constant β = 22°).

Figure 16 and Figure 17 show, respectively, the effects of the lead angle β (Figure 16) and the fiber working length (Figure 17) on the range of fiber influence on surfaces forming an edge. The whiskers shown in the graphs reflect the standard deviation of the results.

## 4. Discussion

The edges of parts are as important as their surfaces because, in general, the weakest element affects the durability of the entire product.

Previous studies of the effects of brushing processes on edge state were mainly related to the analysis of the effects of technological cutting parameters on the condition of a straight edge. This study investigated the effect of a variable lead angle on the condition of a curvilinear edge. The experiments required the use of a five-axis milling machine.

The brushing treatment led to increases in the surface roughness for the blue and white fibers. However, as Matuszak showed in his previous study [46], the effect of brushing on surface roughness strongly depends on the initial roughness. If the initial roughness is high, the appropriate choice of brushing treatment parameters will cut the tops of micro-irregularities and thus reduce the surface roughness. The impact intensity can depend on both the selected tool (diameter, type, and working length of the fibers) and the technological parameters of the brushing process.

It has been observed that if the lead angle is β = 0, the contact conditions between the tool and the workpiece change depending on the curvature of the edge, which affects different chamfer widths along the machined edge. The use of a lead angle of β > 0 ensures a stable and repeatable edge radius regardless of the edge curvature.

As previous studies have shown [35], the brushing process can introduce desirable changes in the surface layer properties. This study investigated the effect of machining conditions on the range of fiber impact on surfaces forming an edge. The changes of the surface layer properties of the surfaces that are close to an edge can have a beneficial effect on the operational life of products exposed to impact phenomena.

## 5. Conclusions

The paper has presented the results of a study investigating the edge states of geometrically complex workpieces that were produced by milling on a multi-axis machining center. The geometrically complex edge samples were made of EN-AW 7075 aluminum alloy. The changes in the edge state after milling were inducted using Xebec tools, specifically ceramic fiber brushes. The effects of the type of brush fibers, fiber working length, and lead angle on the surface roughness and edge state were analyzed. Different brushing strategies were applied on the five-axis machining center. The following results were obtained:

- for the blue (high stiffness) and white (medium stiffness) fibers, the roughness increased after the brushing process compared to the roughness after milling (from Sa = 0.484 µm after milling to Sa = 1.07 µm for the blue and Sa = 0.83 µm for the white fibers);
- for the red (low stiffness) fibers, the roughness remained the same (Sa = 0.46 µm) as after the milling process, which is important in terms of surface quality;
- the brushing treatment led to changes in the arrangement of micro-irregularities (removal of milling marks);
- after the milling process, burrs appeared on the edges, which demonstrates the necessity of using further processing to resolve unfavorable edge states;
- a change in the edge curvature (convex and concave) influenced the change in the contact conditions between the tool and the workpiece for a lead angle of β = 0;
- the use of a lead angle of β = 0 led to a change in the chamfer width along the processed edge, depending on the edge curvature;
- a machining operation conducted with a lead angle of β > 0 ensured a stable and repeatable edge radius regardless of the edge curvature;
- as the fiber working length and the lead angle increased, the range of fiber influence on surfaces forming an edge increased too.

As the fiber working length and lead angle values are increased, the real cutting depth of the fibers in the direction perpendicular to the machined surface also increases as a result of the centrifugal force acting on the flexible fibers. This phenomenon increases the intensity of the impact of the fibers on the machined surface, but it can also cause fiber damage after exceeding the limit value of the immersion. In further studies, a shift of the contact point of the tool at rest (rotational speed = 0) in relation to the tool performing a rotational movement will be analyzed. These studies will be carried out for different rotational speeds and fiber working lengths. This will allow the use of contact point correction on the machine tool, depending on the fiber and the cutting parameters.

## Figures and Tables

**Table 1:** Chemical composition and physical properties of AW 7075-T651 aluminum alloy.

Chemical Composition, wt. %		Physical Properties		Specimen
Cu	1.59	Rm	559 MPa	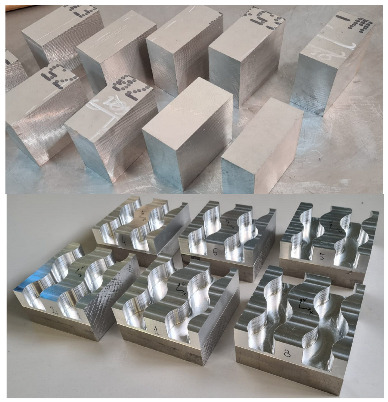
Mn	0.01			
Mg	2.56			
Cr	0.18	Rp_0.2_	448 MPa	
Zn	5.78			
Si	0.07			
Fe	0.13	HB	172	
Ti	0.05			
Al	Rest			

**Table 2:** Lead angle and fiber length values.

Configuration	Lead Angle β (°)	Fiber Working Length W (mm)
	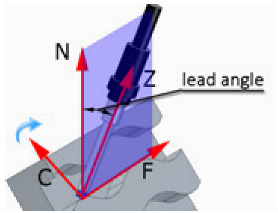	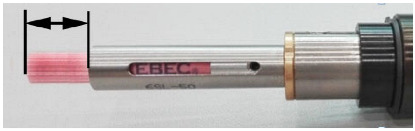
1	15	8
2	15	22
3	29	8
4	29	22
5	12.1	15
6	31.9	15
7	22	5.1
8	22	24.9
9	22	15
10	22	15

**Table 3:** Surface topography after milling and brushing (β = 22°, W = 15 mm).

Sa = 0.484 ± 0.03 µm	Sa = 1.07 ± 0.09 µm
After milling 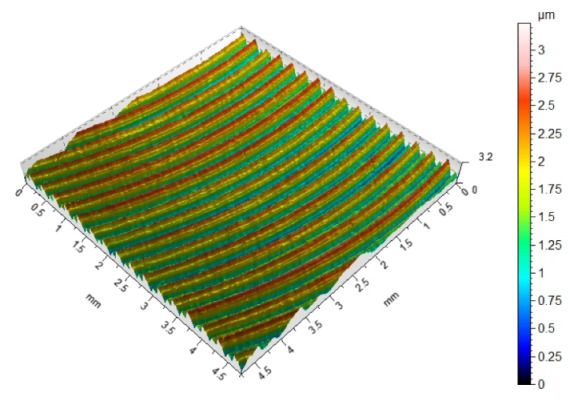	Brushing (blue fiber) 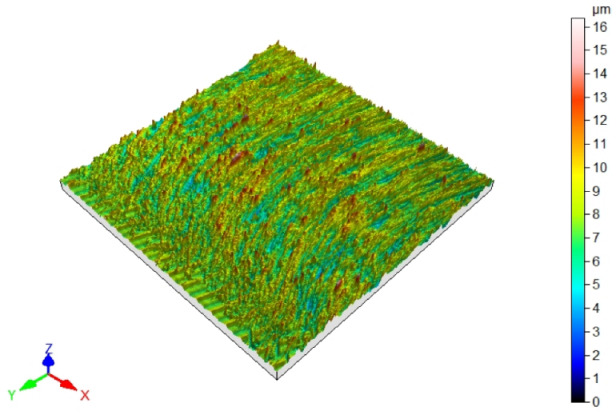
Sa = 0.83 ± 0.11 µm	Sa = 0.46 ± 0.05 µm
Brushing (white fiber) 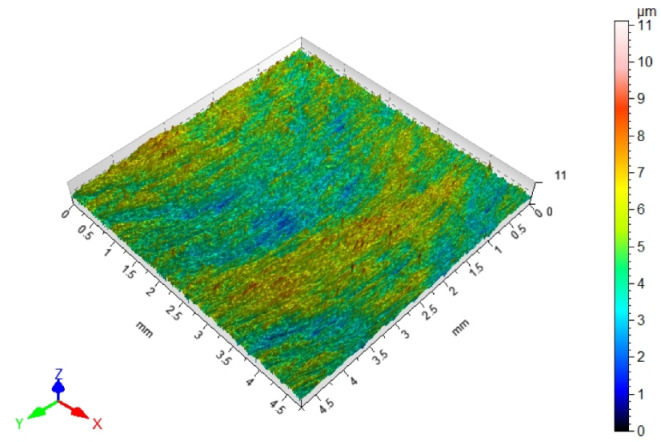	Brushing (red fiber) 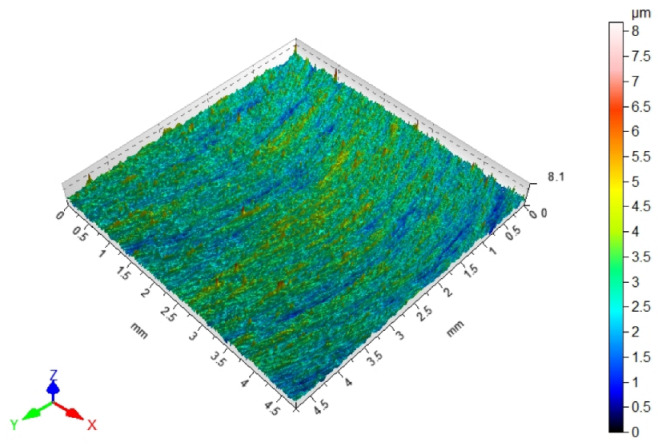

**Table 4:** Edge state after brushing (β = 0°, W = 15 mm).

Convex Edge	Concave Edge	Inflection Point
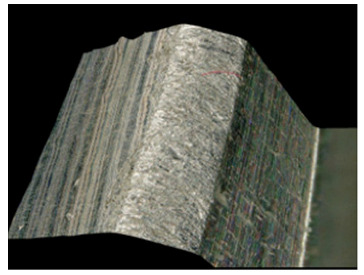	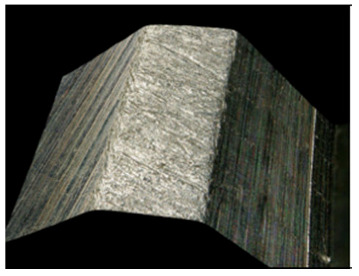	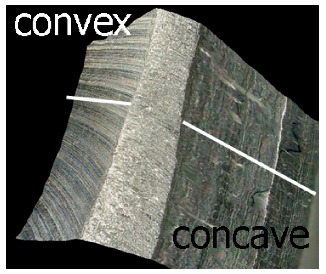
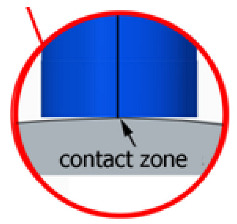	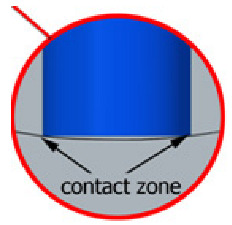	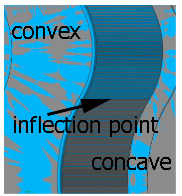

**Table 5:** Edge states after brushing process.

Config.	Lead Angle β (°)	Fiber Working Length W (mm)	Edge State		
			Blue Fiber (High Stiffness)	White Fiber (Medium Stiffness)	Red Fiber Low Stiffness
1	15	8	chamfered	rounded	rounded
2	15	22	chamfered	rounded	rounded
3	29	8	rouned	rounded	rounded
4	29	22	rounded	rounded	rounded
5	12.1	15	chamfered	rounded	rounded
6	31.9	15	rounded	rounded	rounded
7	22	5.1	rounded	rounded	rounded
8	22	24.9	rounded	rounded	rounded
9	22	15	rounded	rounded	rounded
10	22	15	rounded	rounded	rounded

## Data Availability

The original contributions presented in this study are included in the article. Further inquiries can be directed to the corresponding author.
